# Evaluation of learning transfer after a perinatal/neonatal palliative care virtual training course

**DOI:** 10.3389/fped.2023.1215863

**Published:** 2023-06-30

**Authors:** Sinead Brady, Elvira Parravicini, Charlotte Wool

**Affiliations:** ^1^ Department of Pediatrics, Division of Neonatology, Columbia University Irving Medical Center, New York, NY, United States; ^2^ College of Nursing and Health Professions, York College of Pennsylvania, York, PA, United States

**Keywords:** learning transfer, perinatal palliative care, neonatal comfort care, virtual training, training

## Abstract

**Background:**

The success of a training can be determined by the degree of learning transfer. To address a gap in educational offerings during the pandemic, an interdisciplinary team developed and offered a 3-day virtual course, called Next Level Perinatal Palliative Care Training.

**Objective:**

This study aimed to evaluate the transfer of learning and practice from a virtual training course on perinatal/neonatal palliative care (PNPC) by a range of clinicians.

**Study design:**

A descriptive prospective survey design was used to collect data at two time points, immediately following the training course and 6 months later. Frequency and descriptive statistics were used to measure the implementation of PNPC quality indicators, self-reported competence, and clinical facilitators and barriers. A *t*-test was used to compare participants’ anticipated learning transfer to actual learning transfer. Two open-ended items assessed benefits and drawbacks of virtual training.

**Results:**

At course completion, participants anticipated opportunities to implement PNPC strategies with means of 84–87, and at the 6-month mark, the reported implementation had means ranging from 71 to 77. At 6 months post training, participants reported feeling competent/highly competent in each variable with frequency scores of 89%–98%. The opportunity to learn key concepts of PNPC and refresh skill sets ranked as the top facilitators, while the top barriers were the lack of opportunity to use PNPC principles and the lack of funding.

**Conclusion:**

Learning transfer after a virtual training course of PNPC proved to be successful, with a high rate of self-reported actual implementation and competence at 6 months after the training.

## Introduction

Training courses are vital for the dissemination of knowledge and advancement of fields across many industries. Though training courses cannot replace on the job learning, it is a vital tool to help increase one's knowledge, skills, and abilities (1). Within industry, the goal of training is to increase the competence within a targeted area, which ultimately can be leveraged into an organizational benefit (2). In 2012, the American Society for Training and Development found that U.S. Organizations spent $164.2 billion on employee learning and development (3). Given this significant investment, companies have focused on measuring learning transfer with the aim to identify areas that can improve the likelihood that acquired knowledge and skills will be applied to their employees’ daily work (4).

Within the healthcare field, training courses, which historically take place in the form of in-person conferences, play a vital role in continuing medical education (CME). The ultimate beneficiary of successful learning transfer by a clinician is the patient. To that end, to maintain medical licensure, states have different requirements for the amount of CME courses one must complete each calendar year. Due to the COVID-19 Pandemic, in-person training courses were unable to be held, thus many shifted to a virtual platform (5). Though this shift to a virtual platform has been associated with lower travel expenses and easier access to content, some within the healthcare field fear it will lead to less collaborations, decrease in engagement, and decrease in knowledge sharing, with one survey conducted by European Urology showing respondents were less likely to submit abstracts to a strictly virtual conference (5, 6).

Perinatal/neonatal palliative care (PNPC) is a type of care that aims to improve the quality of life of infants when the prolongation of life is no longer the goal of care or the complexity of the medical condition is associated with an uncertain diagnosis (7, 8). PNPC's goal is to maximize the quality of life and comfort of newborns with life-limiting conditions (9). Although there is a growing interest in this field, the training and education in how to deliver this specialized care to this unique population remains limited (10). To address this gap in care, a team at Columbia University Irving Medical Center (CUIMC) created an intensive 3-day in-person training course that improved the self-reported competence of participants across a range of disciplines (11). However, given the pandemic, in 2021 the course was transitioned from an in-person to a virtual format.

Whether the format is in-person or virtual, the success of a training can be determined by the degree of learning transfer and the reach of the course in the number of people able to attend. Transfer of learning is defined as the effective and continuing application of knowledge and skills learned or gained in training to one's practice, with the maintenance of this practice over a period of time (12). In order to assess the success of the virtual training platform for PNPC, this study was created with three specific aims. The study aimed to evaluate the transfer of learning and practice from the virtual PNPC course to everyday practice by a range of clinicians. The study also aimed to better understand the facilitators and barriers to implementation of learnings from the virtual training course. Finally, the study aimed to better understand the benefits and drawbacks of the virtual training platform. Together, this will inform the creation of future training courses to ensure optimal integration of PNPC into practice.

## Methods

### Training course information

To address a gap in educational offerings during the pandemic, a collaborative and interdisciplinary team developed and offered a 3-day virtual intensive training event, called Next Level Perinatal Palliative Care Training. The course was offered in September 2021 and was sponsored by the Departments of Pediatrics and Obstetrics & Gynecology at CUIMC. The training course was synchronous allowing individuals from around the world to join in the event and dialog with one another and the faculty. The training course delivered information via pre-recorded sessions followed by live question and answer sessions with the faculty. Also included were pre-recorded sessions of parent experiences and sessions with clinician's role-modeling strategies to interface with parents during difficult conversations. The curriculum for the training course was built upon PNPC literature, including primary research studies and the eight domains of quality palliative care from the National Consensus Panel (NCP). Details about the training course curriculum were reported previously (11).

### Purpose of the study

Research Aim 1 was to analyze learning transfer 6 months post course to assess participants’ (1) self-reported competence within the eight domains of the NCP and (2) compare anticipated learning transfer to actual learning transfer in select PNPC essentials. Research Aim 2 was to assess facilitators and barriers to implementing PNPC in the clinical setting. Research Aim 3 was to examine benefits and drawbacks of virtual training.

### Study design and statistical analysis

Approval was obtained from the Columbia University Institutional Review Board (IRB-AAAS4060), and informed consent was obtained from participants at the start of data collection. A descriptive prospective survey design was used to collect data at two time points. Qualtrics, an online survey tool, housed the survey that was distributed immediately following the training course and again 6 months following course completion. The survey items included demographic information; seven PNPC learning transfer items adapted from the Centers for Disease Control learning transfer questionnaire (12); nine items on competence, facilitators, and barriers in the clinical setting; and two open-ended items related to the benefits and drawbacks of virtual learning.

Frequency and descriptive statistics were used to measure self-reported competence and clinical facilitators and barriers 6 months post the course. A *t*-test was used to compare participants’ anticipated learning transfer to actual learning transfer for select PNPC essentials. These data were measured on a 0–100 scale, with higher numbers indicating higher degrees of implementation. Krippendorff's content analysis process was applied to examine the participants’ answers to two open-ended items regarding benefits and drawbacks of virtual training.

## Results

The 169 respondents at the end of training were physicians (44%), registered nurses (33%), advanced practice clinicians (12%), and the remaining 11% were social workers, midwives, and those who identified as “other.” Neonatology was heavily represented (62%) followed by palliative care clinicians (18%), “other,” and obstetric providers. The majority (88%) of participants were from the United States (34 states) and 12% came from 18 different countries. Eighty percent of participants had some experience with prenatal or neonatal palliative care consults and actual case experiences (90%). Services are provided in all types of hospitals, including academic medical centers (54%), regional medical centers (18%), and community hospitals (17%). Some participants (12%) provided care in other types of institutions. The 64 respondents to the 6-month survey had similar distribution in terms of demographic and practice characteristics (Table 1).

**Table 1:** Demographic and practice characteristics of respondents.

Characteristics	Respondents immediate post course (*n* = 169)		Respondents 6 months post course (*n* = 64)	
	Total *n*	%	Total *n*	%
Professional status				
Physician	74	43.8%	32	50%
Registered nurse	56	33.1%	20	31.3%
Nurse practitioner/physician assistant	21	12.4%	4	6.3%
Social worker	6	3.6%	2	3.1%
Midwife	2	1.2%	1	1.6%
Other	10	5.9%	5	7.8%
Area of practice				
Neonatology	105	62.1%	43	67.2%
Obstetrics and gynecology	15	8.9%	5	7.8%
Palliative care	31	18.3%	13	20.3%
Other	18	10.7%	3	4.7%
Type of hospital				
Academic medical center	92	54.4%	34	53.3%
Regional medical center	30	17.8%	14	21.9%
Community hospital	28	16.6%	9	14.1%
Other	19	11.2%	7	10.9%
Country of practice				
United States	149	88.2%	52	81.3%
Other	20	11.8%	12	18.8%
Prenatal or neonatal consult experience (years)				
None	33	19.5%	9	14.1%
1–5	49	29%	20	31.3%
6–10	20	11.8%	9	14.1%
11–15	6	3.6%	8	12.5%
Greater than 15	61	36.1%	18	28.1%
Prenatal or neonatal palliative care case experience				
None	16	9.5%	4	6.3%
1–5	42	24.9%	19	29.7%
6–10	23	13.6%	7	10.9%
11–15	13	7.7%	7	10.9%
Greater than 15	75	44.4%	27	42.2%

### Aim 1 results

Nine variables examined self-reported competence at the 6-month mark. Participants reported feeling competent or highly competent in each variable, and frequencies reflected scores between 88.8% and 98.3% (Table 2).

**Table 2:** Self-reported competence 6 months post training (*n *= 64).

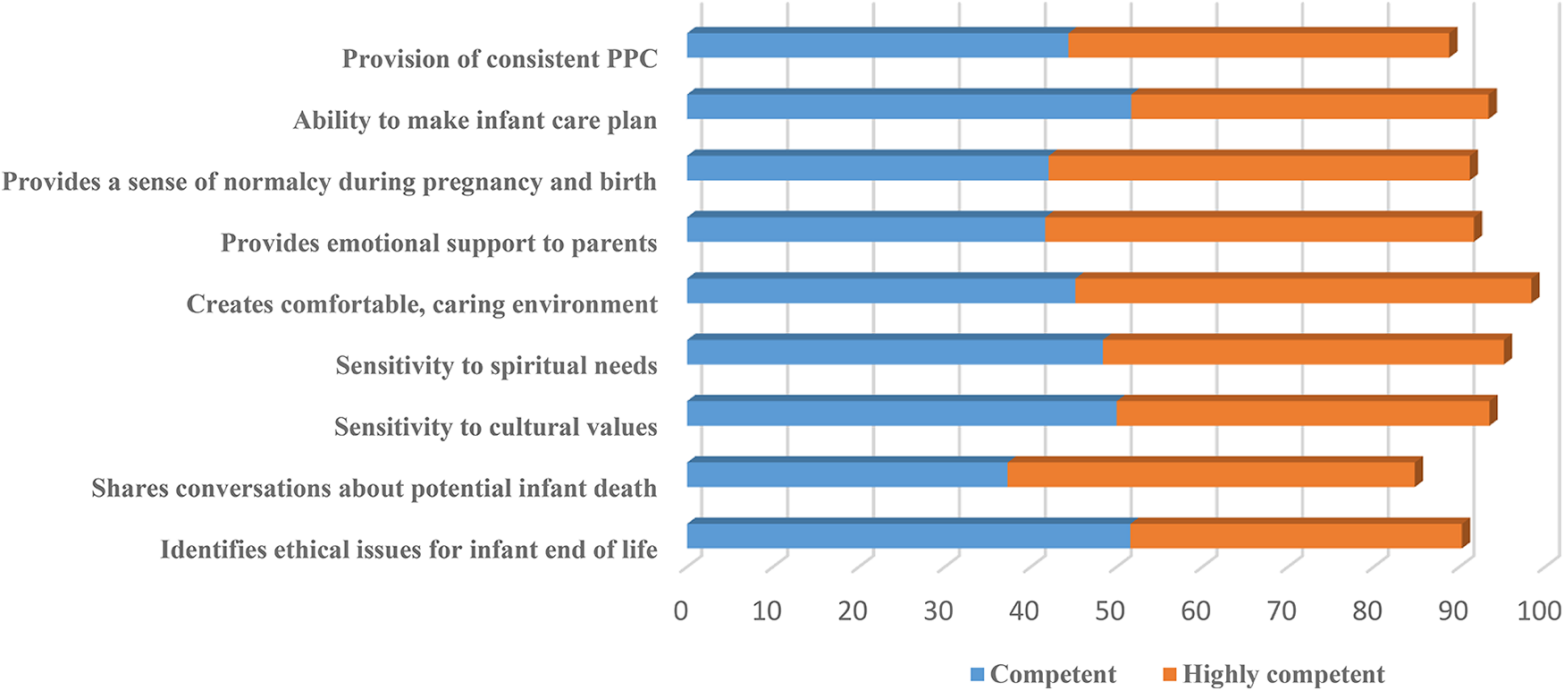

At course completion, participants anticipated opportunities to implement and improve PNPC strategies, with means ranging from 84.1 to 87.5. Actual implementation of measured PNPC variables was statistically significant and lower, with means ranging from 71.1 to 77.3. Only one variable was consistent and that was participants’ motivation to incorporate PNPC into their clinical practice. These data reflected an immediate post-course mean of 97.5 (SD 6.6) vs. a 6-month post-course mean of 95.7 (SD 9.2). (Table 3).

**Table 3:** Comparison of learning transfer of PNPC elements.

	Immediate post course (*n* = 169)		Six months post course (*n* = 64)		
	Mean	SD	Mean	SD	*t*-test; significance
Implementation of new PNPC strategies	85.8	17.9	71.1	23.7	*t* = 4.48; *p* = 0.000
Improvement of evidence-based PNPC	85.9	19.6	73.9	24.3	*t* = 3.53; *p* = 0.001
Improvement communicating with patients	87.5	18.0	77.3	23.3	*t* = 3.15; *p* = 0.002
Incorporation of ethical principles	84.4	20.9	73.6	25.5	*t* = 3.07; *p* = 0.003
Delivery of culturally appropriate care^a^	84.1	20.1	75.1	22.6	*t* = 2.92; *p* = 0.004
Improved level of comfort with consults^a^	85.8	19.8	76.0	23.9	*t* = 3.20; *p* = 0.002
Motivation to incorporate PNPC	97.5	6.6	95.7	9.2	*t* = 1.39; *p* = 0.16

PNPC, perinatal/neonatal palliative care.

^a^ Equal variances assumed.

### Aim 2 results

Facilitators and barriers were ranked by frequency with participants having the option to select all that applied to their clinical area(s). The opportunity to learn key concepts of PNPC and refresh skill sets ranked as the top two facilitators (62% and 53%, respectively), followed by actually using learned skills (50%). Support from supervisors and colleagues were important facilitators as well (42% and 34%, respectively). Barriers in clinical practice were each reported at less than 30%. Most reported barriers were the lack of opportunity to use PNPC principles (29.7%) and the lack of funding (23%). Interestingly, many participants stated they had no barriers to report (29.7%). Time to provide PNPC and support from colleagues and supervisors were still reported as barriers, but at lower frequencies (Table 4).

**Table 4:** Facilitators and barriers six months post course (n = 64).

Participants selected all that applied in each category	
Facilitators	
Reminders of key concepts and skills	62.5%
Opportunity to refresh concepts and skills	53.1%
Opportunities to use learned skills	50%
Support from colleague(s)	42.2%
Support from supervisor(s)	34.4%
Availability of resources needed	32.8%
Time to apply PNPC	26.6%
Barriers	
Lack of opportunities to use what I learned	29.7%
No barriers to report	29.7%
Lack of funding	23.4%
Lack of time	15.6%
Lack of support from colleagues	15.6%
Lack of support from supervisors	14.1%
Lack of confidence	10.9%
Lack of training/education	10.9%
Lack of reimbursement and insurance	9.4%
Lack of professional guidelines	6.3%
Lack of relevance to clinical responsibilities	6.3%

PNPC, perinatal/neonatal palliative care.

### Aim 3 results

The impetus of offering a 3-day training course virtually was the global COVID-19 pandemic. Participants were asked to list three benefits and three drawbacks of virtual training to examine future feasibility of additional virtual course offerings.

The most cited benefit of the virtual platform was cost savings through elimination of travel and lodging expenses and decreased travel time commitment. Global access to training was ranked second with one participant summarizing this concept as “*access to classes I would otherwise not be able to take*.” Participants noted the flexible teaching environment and work–life balance as benefits, with some attending the course from home and others attending while in the clinical setting. Other benefits cited may be unique to this specific training course; specifically, participants appreciated the live question and answer sessions after each topic. The course was recorded and available for several months after it was completed, and participants cited this as a benefit of this virtual training session.

A vast majority of participants reported the inability to network as a disadvantage of the virtual learning environment. One participant summarized the inability to form connections that are so vital in professional conferences by stating a drawback as follows: “*The interpersonal level and relationships formed when in person conferences happen. The connections you make leaving these conferences*.” Distractibility was reported as a limitation with participants who were attending the training during unprotected clinical time. For some, clinical emergencies and/or workload demands interfered with optimal learning. As one participant said, it was “*Easier for ‘the rest of my life’ to ‘intrude during the conference’.*” Participants voiced some disadvantages of technology, ranging from lengthy screen time to loss of focus, as well as minor technology glitches that had to be managed in real time. Of interest, the time zone changes for those participating from outside the course's time zone were both a benefit (course accessibility) and at times a challenge because access to the real-time question and answer sessions. While not frequently reported, some participants missed the hands-on experiential component face-to-face training sessions often offer.

## Discussion

This study demonstrated that an evidence-based multidisciplinary virtual training course of PNPC is associated with a high rate of self-reported implementation and competence at 6 months following the training. Yet, the rate of actual implementation may be limited due to barriers, such as lack of funding or time. Finally, cost-saving and global access vs. interpersonal connection were identified as main benefits and drawbacks of a virtual training course, respectively.

The participants of this study reflect the diverse pool of practitioners who attended the virtual PNPC conference, including physicians, advanced practitioners, nurses and social workers who practice in a variety of settings from community hospitals to academic centers that are located across the world. A core tenet of quality palliative care is the inclusion and representation of an interdisciplinary team (17), which was met with our diverse pool of global participants. Each member of the interdisciplinary team has specific professional skills that benefit patients and families. Furthermore, this range in disciplines, as well as clinical experiences, is important to consider when analyzing our results given there is likely a difference in both personnel and financial resources to pursue or develop PNPC initiatives in one's own institution. Furthermore, there is likely a range in opportunity to practice PNPC based on the acuity and types of cases one's institution handles.

The first aim of this study sought to examine the degree of learning transfer that occurred due to attendance at the virtual conference, which ultimately serves as a proxy to understand if the course material was successfully presented in the virtual format. Six months after the conference, most participants felt competent in all the key areas of PNPC practice (Table 2). This retained competence in PNPC skills 6 months following the course indicates the success of the virtual course in achieving knowledge transfer of the course material. Though participants remained highly motivated to incorporate PNPC into their clinical practice at the 6-month follow-up, actual implementation of PNPC into their practice was lower than what they anticipated after immediate completion of the course (Table 3). Yet, more than 70% of the respondents were able to incorporate PNPC into their practice 6 months following the training. Incorporation of PNPC into clinical practice 6 months after the virtual course mirrors that of participants who attended the in-person conference organized by the same multidisciplinary team at CUIMC in 2019 (13). These findings point to the fact that attendance of evidence-based educational opportunities both virtual and in-person increases the clinicians’ ability to translate PNPC into their clinical practice.

Understanding some of the facilitators and barriers to implementing PNPC into practice offers insight as to why not all respondents were able to fully integrate what they learned into their clinical care, despite feeling competent in the material.

The main barrier to utilizing PNPC is the lack of opportunity with many respondents indicating they had yet to have a clinical scenario that required them to utilize these skills. Lack of funding to initiate and or develop a PNPC program or provide care was also noted as a barrier. Funding is a common barrier noted in the literature (14), which may be addressed carefully and systematically over time. Champions for palliative care initiatives can consider carefully describing comfort/palliative care to colleagues and administrators, working with administration and outside funding sources, or participating in institution-wide committee work to explore avenues to maximize resources (15). Finally, support from colleagues and time to provide PNPC were listed as further barriers. Interestingly, respondents identified a larger presence of facilitators (62.5%–26.6%) than barriers (29.7%–6.3%) and one-third reported no barriers at all.

The opportunity to learn key concepts and refresh skills and being able to use the learned skill in a clinical setting were reported as the top facilitators to incorporating PNPC into clinical practice. One's opportunity to utilize PNPC likely will vary based on their role within the clinical team as well as their practice setting. Therefore, it is important that providers have tools that will allow them to maintain their skillsets, despite not having regular opportunities to practice these skills clinically.

Although PNPC is a growing field, training and education in delivering evidence-based information to interdisciplinary team members is limited. Unlike other disciplines where there are multiple conferences and educational opportunities across the world for practitioners to obtain new skills or stay up to date on the latest trends in the field, to our knowledge, this is the only PNPC dedicated course. Many healthcare providers have expressed interest in additional training in delivering PNPC, with one survey of over 400 providers in Sweden indicating that they would like more training (14). Furthermore, institutions who would like to have the principles of neonatal palliative care taught to their staff or students have had difficulty finding people qualified to teach this material (16, 17). One study found that more than half of NICUs do not have any comfort care guidelines, and of those surveyed, 91% noted that their institution would benefit from additional PNPC education (18). This aligns with nationally recognized organizations including Worldwide Palliative Care, National Consensus Project for Quality Palliative Care, and American College of Obstetrics and Gynecology, all of whom have recommendations that institutions develop and implement PNPC programs (9, 19, 20). This virtual conference addresses the significant need for increased education in PNPC noted within the neonatology community, which is ultimately needed before any formal program can be established. Furthermore, our participants agreed that broader access to the training conference through a virtual platform was a primary benefit.

The elimination of travel costs and time to travel was noted as the main benefit to the virtual training course. Other virtual training courses have also noted this to be a benefit for their attendees, allowing their training course to reach more individuals from around the world (21). This is also reflected in our data, in that we had attendees from 31 states and 14 different countries represented at the conference. The recording of the virtual course that was made available to attendees for several months after the course was also a noted benefit. Again, this is a common noted benefit for other virtual conferences (21). In recording and developing the virtual course, our team partnered with a video production company, which ensured that the video quality and the virtual training course platform optimized the experience for attendees. Furthermore all recordings and interactive sessions were filmed from the same central location; thus, issues with speakers’ internet connection or inability to log on to the conference were able to be managed in real time by the on-site IT team. This mitigated conference disruptions that can occur when speakers are left to troubleshoot issues on their own (21). The main drawback that respondents highlighted was the lack of opportunity to network. This too has been a noted issue for other virtual courses (6). For future course design, providing opportunities for networking during a virtual course should be addressed. Though the course had live question and answer sessions, perhaps having smaller breakout rooms where small groups can discuss topics will allow for more opportunity to network.

This study has several strengths. The participants in both the initial and 6-month follow-up survey reflect a diverse pool of practitioners both in terms of specialties as well as practice setting and location. Because of this diversity in the respondent pool, we can be confident that the findings that learning transfer can successfully occur via a virtual conference can be broadly applied. Additionally, the timing of the second survey as a 6-month follow-up allowed for the measurement of integration of PNPC into clinical practice, something our group has measured previously but in a live training course (13).

This study also has limitations. There were 64 participants who responded to the 6-month follow-up survey, which represents only 38% of the initial post-course responses. The low response rate at the 6-month follow-up compared to the initial survey is likely due to the respondents receiving their CME certificate upon completion of the first survey. Though we attempted to motivate respondents to complete the 6-month follow-up survey with multiple email reminders and the chance to win a gift card, we were unable to generate enough of a drive to come back and complete the survey. Nevertheless, the diversity of the responders at the 6-month mark adds confidence to the findings. This study assesses self-reported competence in areas of PNPC, but not actual skills assessed by a third-party observer. Finally, self-reported learning transfer compared with evaluations made by colleagues or superiors has been reported to likely be more positive (22).

In conclusion, learning transfer after a virtual training course of PNPC proved to be successful, with a high rate of self-reported actual implementation and competence 6 months after the training. The difference between the anticipated and actual rate of implementation may be due to barriers. A virtual training platform provides global access and is a useful tool to help further expand the field of PNPC.

## Data Availability

The raw data supporting the conclusions of this article will be made available by the authors, without undue reservation.
